# Effects of Age, Gender and Laterality on the sEMG of the Orbicularis Oculi in Healthy Adults

**DOI:** 10.3390/jcm14093119

**Published:** 2025-04-30

**Authors:** Larysa Krajewska-Węglewicz, Małgorzata Dorobek

**Affiliations:** 1 Department of Ophthalmology, National Institute of Medicine of the Ministry of Interior and Administration, 02-507 Warsaw, Poland; 2 Department of Neurology, National Institute of Medicine of the Ministry of Interior and Administration, 02-507 Warsaw, Poland

**Keywords:** orbicularis oculi muscle, surface electromyography, gender differences, laterality, aging

## Abstract

**Background/Objective**: The orbicularis oculi muscle (OOM) is crucial for eyelid function and ocular protection. Surface electromyography (sEMG) is a useful tool for assessing OOM activity, but interpretation remains limited due to a lack of standardized reference values. This study investigates the influence of gender, laterality, and age on OOM activity using sEMG. **Methods**: In total, 84 healthy adult participants (44 females and 40 males) underwent sEMG measurements for both eyes during gentle (GEC) and maximal (MVC) voluntary eyelid closure. **Results**: The average age was 62.5 years (ranging from 27 to 86; SD = 13.79) for males, and 63.12 years (ranging from 27 to 87; SD = 13.8) for females. The mean Root Mean Square (RMS) MVC values were 157.80 ± 56.43 for men and 146.45 ± 56.48 for women. The mean RMS GEC values were 7.42 ± 2.94 for men and 8.35 ± 4.86 for female. No significant gender-based differences were found (*p*-value = 0.195 and 0.138, respectively). No significant differences between sides were found: The mean RMS MVC values were 152.24 ± 57.73 for left eyes and 151.47 ± 55.74 for right eyes (*p*-value = 0.93). The mean RMS GEC values were 8.29 ± 5.04 for left eyes and 7.53 ± 2.79 for right eyes (*p*-value = 0.227). The age of participants correlated negatively with maximal amplitude, mean amplitude, RMS and the difference between RMS and RMS baseline in MVC (*p*-value < 0.001). **Conclusions**: While OOM function remains consistent across gender and between eyes, aging contributes to a decline in OOM performance. Age-adjusted reference values may be beneficial in clinical and research applications assessing facial muscle function.

## 1. Introduction

The orbicularis oculi muscle (OOM) is an essential component of facial musculature, playing a pivotal role in eyelid function and facial expression. It facilitates both voluntary and involuntary movements, including spontaneous blinking, gentle eyelid closure, and forceful eye closure [1]. It facilitates tear drainage through rhythmic contraction during blinking [2]. Functionally, the OOM is vital for ocular protection, as it maintains corneal hydration, prevents the intrusion of foreign bodies, and promotes the even distribution of the tear film [3]. Impairments in OOM function are associated with a range of clinical conditions, such as Bell’s palsy, blepharospasm, facial nerve paralysis, and neuromuscular disorders including amyotrophic lateral sclerosis and Parkinson’s disease [4,5,6]. Moreover, age-related degeneration of the OOM contributes to functional and esthetic eyelid disorders, such as lagophthalmos and dermatochalasis, with potential consequences for visual acuity and ocular surface health.

Surface electromyography (sEMG) is a widely used, non-invasive technique for assessing muscle activity by detecting electrical signals generated during muscle contractions [7]. While sEMG has been extensively applied in neuromuscular research and clinical diagnostics, the interpretation of OOM activity remains limited by a lack of standardized reference values. Several demographic and physiological factors, including gender, side dominance (laterality), and aging, may influence sEMG recordings and require further investigation.

The assumption of bilateral equivalence is crucial for clinical assessments and research studies that rely on unilateral sEMG measurements as a proxy for overall neuromuscular function. On the one hand, the assumption of symmetrical OOM activity may facilitate the development of standardized rehabilitation protocols. On the other hand, the presence of significant lateral differences would necessitate the consideration of factors such as ocular dominance or muscular asymmetry in the interpretation of sEMG findings.

Sex-related differences in neuromuscular performance have been well documented across various skeletal muscles, yet data specific to the OOM are sparse [8,9]. Hormonal and structural distinctions between males and females may influence muscle fiber composition, activation dynamics, and fatigue resistance [10]. Clarifying whether OOM activity varies by sex is therefore crucial for determining the necessity of sex-specific normative sEMG values.

Aging is associated with progressive declines in neuromuscular function due to factors such as motor unit remodeling, reduced muscle mass, and altered neuromuscular control. Studies have shown that the OOM undergoes structural changes with age, including a decrease in muscle thickness and an increase in orbital fat prolapse, which may contribute to diminished eyelid closure strength [11]. Additionally, electromyographic studies on other facial muscles have reported reductions in muscle activation with aging, but data specific to the OOM remain limited. Establishing how aging influences OOM sEMG activity is essential for distinguishing normal age-related changes from pathological conditions such as facial nerve palsy or blepharospasm.

The objective of this study is to evaluate the influence of gender and laterality on sEMG parameters using RMS-MVC and RMS-GEC values, and to analyze the effect of age on these measurements.

## 2. Materials and Methods

### 2.1. Participants

In total, 84 healthy subjects aged 27–87, 44 females and 40 males, participated in the study. Participants were divided by gender, and sEMG measurements were collected from both eyes.

Participants were excluded if they had any neurological conditions involving the neuromuscular junction, broader neurological disorders, or systemic illnesses with potential neurological complications, such as diabetes mellitus. Individuals with a family history of neuromuscular diseases were also excluded. Additional exclusion criteria included the use of medications influencing peripheral nervous system function, a history of substance abuse or alcohol dependency, and prior administration of botulinum toxin in any muscle. Subjects were also excluded if they had dermatological conditions affecting the eyelid skin, blepharochalasis, a history of trauma or surgical procedures involving the eyelids, current use of medications known to alter skin or muscle structure, and active smoking.

### 2.2. Electrode Placement

**Figure 1:**
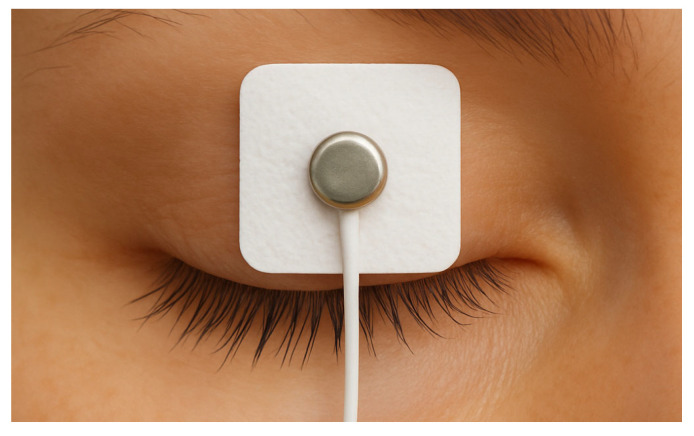
Schematic position of the active electrode.

Surface electrodes were applied as follows: the active electrode was placed horizontally across the central portion of the upper eyelid (Figure 1), approximately 5 mm above the line of the eyelashes; the reference electrode was positioned on the forehead, while the ground electrode was secured over the left clavicle.

### 2.3. sEMG Measurement Protocol

Participants remained seated in an upright posture with their heads maintained in a neutral alignment throughout the procedure. Electromyographic signals were recorded under three conditions: (1) maximal voluntary contraction (MVC), achieved through deliberate and strong eyelid closure; (2) gentle eyelid closure (GEC), reflecting light muscular activation; and (3) resting state, recorded with the eyes open and focused in primary gaze.

**Figure 2:**
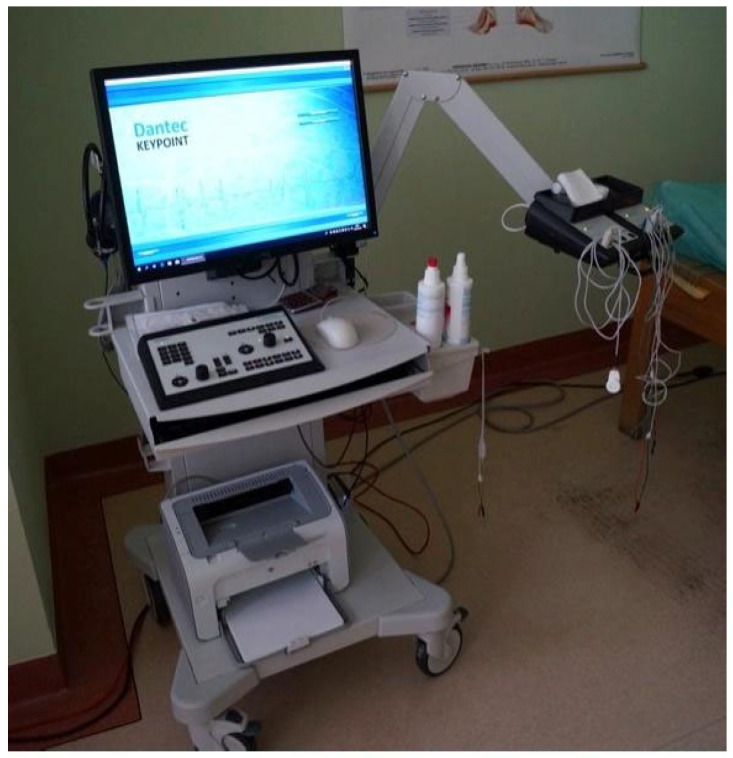
Outpatient setting. Dantec Keypoint EMG workstation.

Each condition was recorded over a five-second interval, and three consecutive trials were performed. To ensure sufficient muscular recovery and reduce the potential for fatigue, a one-minute rest period was observed between trials. For each condition, the average of the three measurements was calculated and used for further analysis (Figure 2).

### 2.4. Ethics Approval

This study involved human participants and received approval from the Ethics Committee of the National Institute of the Ministry of Interior and Administration (Approval No. 45/2024). All procedures adhered to the principles outlined in the Declaration of Helsinki. Written informed consent was obtained from all participants prior to their inclusion in this study.

### 2.5. Statistical Analysis

Independent samples *t*-tests assessed gender and laterality differences. Pearson correlation and regression analyses evaluated the relationship between age and sEMG parameters. The calculations were performed with the use of reflimR package working in R Statistics 4.3.3 open-source software environment. The qualitative findings were summarized narratively.

## 3. Results

A total of 84 individuals, ranging in age from 27 to 87 years (mean age = 63.12, SD = 13.80), were enrolled in the study. The sample included 44 female participants (aged 27–87, M = 63.12, SD = 13.80) and 40 male participants (aged 27–86, M = 62.50, SD = 13.79). Data were collected bilaterally from each participant, resulting in a total of 168 eye-specific observations.

All procedures were well tolerated, and no participants reported experiencing discomfort during the recordings.

### 3.1. Differences Between Males and Females

Table 1 depicts the mean values of the analyzed variables in the group females and in the group of males with the values of independent samples Student’s *t* test.

No statistically significant differences between males and females were detected.

### 3.2. Differences Between the Values for the Left and the Right Eye

Table 2 displays the average values of the examined parameters for the left and right eyes, accompanied by the corresponding *p*-values from the independent samples Student’s *t*-test assessing differences between sides.

No statistically significant differences between the values for the left eye and the values for the right eye were detected.

### 3.3. Age of Participants

The Pearson correlation coefficients between participants’ age and the analyzed variables are summarized in Table 3.

The age of participants correlated negatively with maximal amplitude, mean amplitude, RMS and the difference between RMS and RMS baseline in maximal voluntary contraction (MVC).

### 3.4. The Differences Between Males and Females, Between the Left and the Right Eye and the Age of Participants as Predictors of Analyzed Variables

The differences between males and females, between the left and the right eye and the age of participants as predictors of analyzed variables with the use of regression analysis based on the enter method. Each variable was analyzed in a separate regression model. The results are depicted in Table 4.

**Figure 3:**
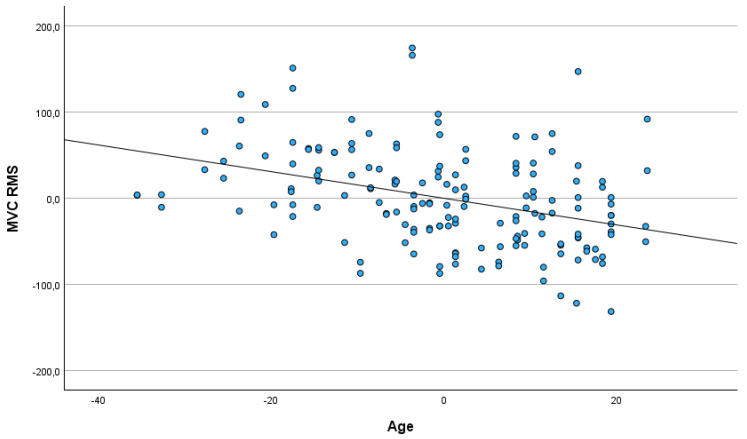
Partial regression plot depicting relationship between participants’ age and RMS in maximal voluntary contraction (MVC).

**Figure 4:**
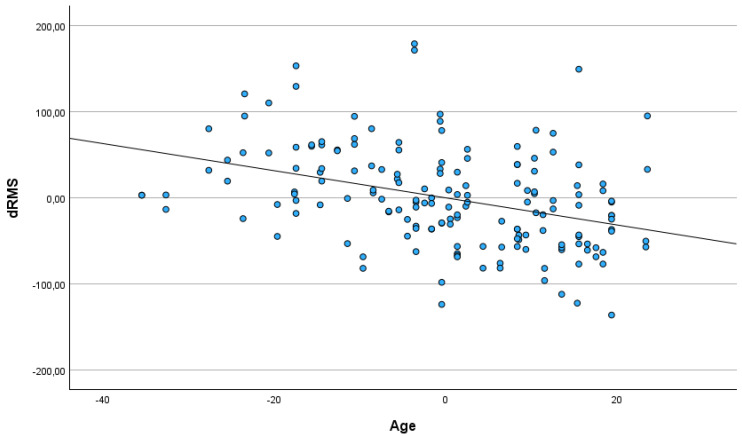
Partial regression plot depicting relationship between participants’ age and the difference between RMS and RMS baseline in maximal voluntary contraction (MVC).

The age of participants related negatively to maximal amplitude, mean amplitude, RMS and the difference between RMS and RMS baseline in MVC. No statistically significant association between participants’ sex or the differences between the left and right eye were detected. The older participants had lower values of ampl. max, ampl. mean, RMS and the difference between RMS and RMS baseline in maximal voluntary contraction (see Figure 3 and Figure 4).

## 4. Discussion

This study evaluated the effect of aging on sEMG measurements of the OOM and investigated potential differences in sEMG results of the OOM between men and women and between the left and right side.

### 4.1. Age-Related Decline in Neuromuscular Function

Age was found to be inversely related to MVC-related parameters—namely, maximum amplitude, mean amplitude, and RMS values. This relationship is in line with earlier studies of other skeletal muscles that have documented a decline in neuromuscular function and muscle strength with advancing age. Piasecki, when comparing the vastus lateralis and tibialis anterior in young and old participants, found that older males had fewer but larger motor units. Additionally, these older motor units exhibited greater instability in neuromuscular transmission and lower firing rates, indicating a decline in neuromuscular function with age [12]. Age-related decrement in the sEMG response is indicative of a generalized decline in the motor performance of the OOM. These reductions are likely due to age-associated changes in motor unit recruitment, shifts in muscle fiber composition, and alterations in neuromuscular control.

The findings of the present study are consistent with our earlier research conducted on a separate cohort, in which we observed that patients with blepharochalasis over the age of 60 exhibited significantly reduced OOM activity during maximal voluntary contraction (MVC) compared to younger individuals. This age-related decline was evident in both the mean amplitude (*p* = 0.029) and RMS values (*p* = 0.045). Notably, ultrastructural analysis revealed morphological features characteristic of skeletal muscle aging within the OOM, irrespective of the participants’ age, suggesting that structural degeneration may precede or occur independently of functional decline [11]. In the present study, individuals with known neurological conditions were excluded. However, the potential influence of other systemic conditions that may affect muscle tone was not examined. Future research should aim to address this limitation by systematically evaluating the role of comorbidities in neuromuscular function.

Importantly, the age-related decline in OOM function observed with surface EMG has also been supported by findings from needle EMG studies. Needle EMG allows for direct recording of individual motor unit potentials, offering insight into motor unit number and firing characteristics. Studies employing needle EMG in aging populations have demonstrated similar patterns—such as increased motor unit potential duration and polyphasia, reduced recruitment, and decreased firing rates—consistent with motor unit remodeling and denervation–reinnervation processes that accompany aging. While needle EMG provides more detailed motor unit-level information, sEMG offers a non-invasive and functionally relevant assessment of muscle performance during natural movements such as blinking or voluntary eye closure. The convergence of findings from both techniques reinforces the evidence of age-related neuromuscular decline in periorbital muscles like the OOM [13].

Okuda et al. in a cross-sectional study involving both men and women revealed that older individuals exhibit a thinner OOM and a greater degree of orbital fat prolapse compared to younger subjects, with these two variables showing a negative correlation [14]. In this study, computed tomography was used to measure the OOM thickness in 34 adults aged between 20 and 79 years. The results indicated a significant reduction in muscle thickness with age.

Conversely, Fukuda and Kajiya [15] reported that while the mean myofiber cross-sectional area of the OOM did not differ significantly between young and older adults, the proportion of the muscle area composed of myofibers was lower in the older group. It is worth noting that in the study by Fukuda and Kajiya, the specific region measured was not detailed, and the exact age ranges defining the young and old groups (within the overall sample aged 19 to 62 years) were not provided.

In addition, in a study on blinking it has been reported that the percentage of incomplete eye closure increases in older adults (range 51–77 years) compared with young adults (range 20–30 years) [16].

Together, these results suggest that the function of OOM is affected by age and that change may be due to decreases in strength. Consequently, incorporating age-adjusted reference values into clinical assessments is advisable.

### 4.2. Gender Differences in Neuromuscular Function

In skeletal muscles differences between men and women in absolute strength in various muscle groups have been reported [17,18,19]. However, we found no statistically significant differences between male and female participants across any of the analyzed variables in OOM. Similarly, Bertozzi et al. found no statistically significant difference between male and female participants in the sEMG study of facial muscles that included OOM [20]. Kim et al. investigated RMS values of facial muscles as well, and reported no significant gender differences. However, their study of facial muscles did not include an analysis of the OOM [21]. This contrasts with findings from ultrasound studies, which have reported that OOM thickness is approximately 16% smaller in women than in men [22]. However, it is possible that muscle thickness differences do not translate directly to differences in sEMG amplitude or activation patterns. Additionally, Volk et al. observed an age-related decline in OOM size in men but not in women in ultrasound measurements, suggesting that sex differences in OOM function may become more apparent with aging [23].

In a study of lid power assessment, including 50 participants, based on the analysis of the maximum compression of the lid speculum, a weak correlation was found between the sex of the subjects and eyelid power [24].

Altogether, the influence of gender on OOM function needs further investigation, but it seems that there is no evident predominance of neither the sex.

### 4.3. Laterality: Left vs. Right Eye Measurements 

Our analysis did not reveal significant differences between left and right eye measurements, indicating symmetry in neuromuscular function, which is consistent with previous study of Bertozzi et al. [20]. The study of Mueller et al. also noted that there was no significant difference between the left and right sides of the face in terms of muscle activation patterns, indicating a symmetrical activation during the tasks [25]. The issue of muscle symmetry in facial palsy has been addressed in a systematic review by Franz et al. By introducing an ‘asymmetry index’ in sEMG assessments, the authors aim to eliminate side predominance, using the comparison between the healthy and affected sides as the basis for normalization [26]. Volk et al. demonstrated in a MRI study on facial muscles volume that in healthy individuals the volume of the orbicularis oculi muscle is symmetrical, further supporting the assumption that neuromuscular function is bilaterally equivalent [27].

## 5. Conclusions

The results of the present study demonstrated a significant effect of age on the sEMG performance of the OOM and suggested that this variable should be taken into consideration in the interpretation of OOM sEMG in basic and clinical studies. No significant differences were observed between genders or between left and right side.

## Figures and Tables

**Table 1:** Mean values of the analyzed variables in the group females and in the group of males.

		Females		Males				
	Variable	M	SD	M	SD	t	df	*p*
	RMS baseline	11.20	5.28	10.23	5.81	1.13	166	0.261
MVC	ampl. max	1197.26	411.63	1283.51	449.58	−1.30	166	0.196
	ampl. Mean	349.37	91.03	369.67	95.44	−1.41	166	0.160
	RMS	146.45	56.48	157.80	56.43	−1.30	166	0.195
	ΔRMS	135.25	57.32	147.56	58.00	−1.38	166	0.169
GEC	ampl. max	52.15	23.15	49.26	19.40	0.87	166	0.385
	RMS	8.35	4.86	7.42	2.94	1.49	166	0.138
	ΔRMS	−2.84	6.85	−2.81	5.39	−0.03	166	0.974

M—mean value; SD—standard deviation; t—value of Student’s *t* test for independent samples; df—degrees of freedom; *p*—two-tailed statistical significance.

**Table 2:** Mean values of the analyzed variables for the left eye and the right eye.

		Left		Right				
	Variable	M	SD	M	SD	t	df	*p*
	RMS baseline	10.40	6.39	11.08	4.56	−0.80	166	0.426
MVC	ampl. max	1221.26	448.77	1255.41	414.44	−0.51	166	0.609
	ampl. Mean	358.38	96.45	359.69	90.89	−0.09	166	0.928
	RMS	152.24	57.73	151.47	55.74	0.09	166	0.930
	ΔRMS	141.84	59.22	140.39	56.70	0.16	166	0.871
GEC	ampl. max	52.35	22.16	49.19	20.70	0.96	166	0.340
	RMS	8.29	5.04	7.53	2.79	1.21	166	0.227
	ΔRMS	−2.11	7.47	−3.55	4.47	1.52	166	0.130

M—mean value; SD—standard deviation; t—value of Student’s *t* test for independent samples; df—degrees of freedom; *p*—two-tailed statistical significance.

**Table 3:** Correlation coefficients between the age of participants and analyzed variables.

		Age	
	Variable	r	*p*
	RMS baseline	0.073	0.344
MVC	ampl. max	−0.344	0.001
	ampl. Mean	−0.358	0.001
	RMS	−0.380	0.001
	ΔRMS	−0.379	0.001
GEC	ampl. max	−0.066	0.393
	RMS	−0.018	0.813
	ΔRMS	−0.078	0.315

r—Pearson correlation coefficient; *p*—two-tailed statistical significance.

**Table 4:** The differences between males and females, between the left and the right eye and the age of participants as predictors of analyzed variables.

	Variables	Predictors	Beta	t	*p*	F	df	p	R^2^
MVC	ampl. max	Males vs. females	0.09	1.17	0.243	7.96	3164	0.001	0.127
		Eye left vs. right	0.04	0.55	0.587				
		Age	−0.34	−4.66	0.001				
	ampl. mean	Males vs. females	0.09	1.29	0.199	8.68	3164	0.001	0.137
		Eye left vs. right	0.01	0.10	0.923				
		Age	−0.35	−4.88	0.001				
	RMS	Males vs. females	0.08	1.17	0.244	9.75	3164	0.001	0.151
		Eye left vs. right	−0.01	−0.10	0.925				
		Age	−0.38	−5.23	0.001				
	ΔRMS	Males vs. females	0.09	1.26	0.210	9.79	3164	0.001	0.152
		Eye left vs. right	−0.01	−0.18	0.861				
		Age	−0.38	−5.21	0.001				
GEC	ampl. max	Males vs. females	−0.07	−0.91	0.365	0.08	3164	0.483	0.015
		Eye left vs. right	−0.07	−0.96	0.341				
		Age	−0.07	−0.90	0.372				
	RMS	Males vs. females	−0.12	−1.50	0.136	1.26	3164	0.291	0.022
		Eye left vs. right	−0.09	−1.21	0.227				
		Age	−0.02	−0.30	0.762				
	ΔRMS	Males vs. females	0.00	−0.01	0.991	1.11	3164	0.349	0.020
		Eye left vs. right	−0.12	−1.52	0.131				
		Age	−0.08	−1.01	0.315				

Beta—standardized regression coefficient; t—the value of statistical test for a predictor; *p*—statistical significance; F—the value of the statistical test for a model; df—degrees of freedom; R^2^—determination coefficient.

## Data Availability

All data relevant to the study are included in the article.

## Abbreviations

The following abbreviations are used in this manuscript:

OOM	orbicularis oculi muscle
sEMG	surface electromyography
MVC	maximal voluntary contraction
GEC	gentle eyelid closure
